# *Mastomys natalensis* (Smith, 1834) as a natural host for *Schistosoma haematobium* (Bilharz, 1852) Weinland, 1858 x *Schistosoma bovis* Sonsino, 1876 introgressive hybrids

**DOI:** 10.1007/s00436-021-07099-7

**Published:** 2021-03-09

**Authors:** Boris A.E.S. Savassi, Gauthier Dobigny, Jonas R. Etougbétché, Thalasse T. Avocegan, François T. Quinsou, Philippe Gauthier, Moudachirou Ibikounlé, Hélène Moné, Gabriel Mouahid

**Affiliations:** 1 University of Perpignan Via Domitia, IHPE UMR 5244, CNRS, IFREMER, University of Montpellier, 58 Avenue Paul Alduy, Bât. R, F-66860 Perpignan, France; 2 Faculté des Sciences et Techniques, Université d’Abomey-Calavi, 01BP526 Cotonou, Benin; 3 Ecole Polytechnique d’Abomey-Calavi, Laboratoire de Recherche en Biologie Appliquée, Université d’Abomey-Calavi, Cotonou, Benin; 4 Institut de Recherche pour le Développement, Centre de Biologie et de Gestion des Populations (UMR IRD/INRA/CIRAD/Institut Agro), Montferrier-sur-Lez, France

**Keywords:** *Schistosoma haematobium* x *Schistosoma bovis*, Cercarial chronobiology, *Mastomys natalensis*, *Rattus rattus*, Schistosome transmission

## Abstract

Cercarial emission of schistosomes is a determinant in the transmission to the definitive host and constitutes a good marker to identify which definitive host is responsible for transmission, mainly in introgressive hybridization situations. Our goal was to test the hypothesis that micro-mammals play a role in *Schistosoma haematobium*, *S*. *bovis*, and/or *S*. *haematobium* x *S*. *bovis* transmission. Small mammal sampling was conducted in seven semi-lacustrine villages of southern Benin. Among the 62 animals trapped, 50 individuals were investigated for *Schistosoma* adults and eggs: 37 *Rattus rattus*, 3 *Rattus norvegicus*, 9 *Mastomys natalensis*, and 1 *Crocidura olivieri*. *Schistosoma* adults were found in four *R. rattus* and two *M. natalensis*, with a local prevalence reaching 80% and 50%, respectively*.* Two cercarial chronotypes were found from *Bulinus globosus* experimentally infected with miracidia extracted from naturally infected *M. natalensis*: a late diurnal and nocturnal chronotype, and an early diurnal, late diurnal, and nocturnal chronotype. The cytochrome C oxidase subunit I mtDNA gene of the collected schistosomes (adults, miracidia, and cercariae) belonged to the *S*. *bovis* clade. Eleven internal transcribed spacer rDNA profiles were found; four belonged to *S*. *bovis* and seven to *S*. *haematobium* x *S*. *bovis*. These molecular results together with the observed multi-peak chronotypes add *M. natalensis* as a new host implicated in *S*. *haematobium* x *S*. *bovis* transmission. We discuss the origin of the new chronotypes which have become more complex with the appearance of several peaks in a 24-h day. We also discuss how the new populations of offspring may optimize intra-host ecological niche, host spectrum, and transmission time period.

## Introduction

Schistosomiasis is an acute and chronic disease caused by flatworms of the genus *Schistosoma* and estimates have shown that at least 229 million people required preventive treatment in 2018 (WHO 2020). The lifecycle of schistosomes is complex and includes a vertebrate definitive host, where the adult schistosomes pair and reproduce, and a gastropod snail intermediate host where the larvae multiply asexually. Transmission from the vertebrate host to the snail host is ensured by the miracidium larva. Transmission from the snail host to the vertebrate host is ensured by the cercaria larva which actively penetrates the vertebrate host. The timing of *Schistosoma* cercarial shedding from the intermediate snail hosts is synchronized by exogenous factors (the major synchronizer is the photoperiod) and is highly correlated to the time interval when the definitive host goes to water (Combes et al. 1994). As such, cercarial emission of schistosomes is a primordial parameter that determines successful transmission to the definitive mammalian host. As examples, the cercariae of *Schistosoma bovis* Sonsino, 1876 are shed during the early morning when cattle go to watering (Mouahid and Théron 1986; Mouahid et al. 1987, 1991); the cercariae of *Schistosoma haematobium* (Bilharz, 1852) Weinland, 1858 and *S. mansoni* Sambon, 1907 are shed around mid-day when humans go to water for bathing, washing, or fishing (Mouahid et al. 1991, 2012); finally, the cercariae of *S. rodhaini* Brumpt 1931 and of some chronotypes of *S. mansoni* and of *S. japonicum* Katsurada 1904 are shed during the early night, when nocturnal rodents actively use water (Théron 1989; Mouahid et al. 2012; Su et al. 2013). This makes cercarial pattern a good marker for identifying which definitive host is responsible for *Schistosoma* transmission, mainly in the introgressive hybridization situations between different species of schistosomes that have increasingly been described in the literature over the last decade (Boissier et al. 2019).

Savassi et al. (2020) showed, for the first time, that cattle could be responsible for the transmission of *S. haematobium* x *S. bovis* populations in Benin, West Africa. The authors found atypical cercarial chronotypes: (i) early shedding that may correspond to *S. bovis*, (ii) mid-day shedding that may correspond to *S. haematobium*, and (iii) nocturnal cercarial emission unknown for the two species. The latter nocturnal chronobiological profile for *S. haematobium* x *S. bovis* raises suspicion that nocturnal mammals, such as rodents, could play a role in the transmission. The goal of this study is to test the hypothesis that micro-mammals play a role in schistosome transmission.

## Materials and methods

### Sites of collection

**Fig. 1:**
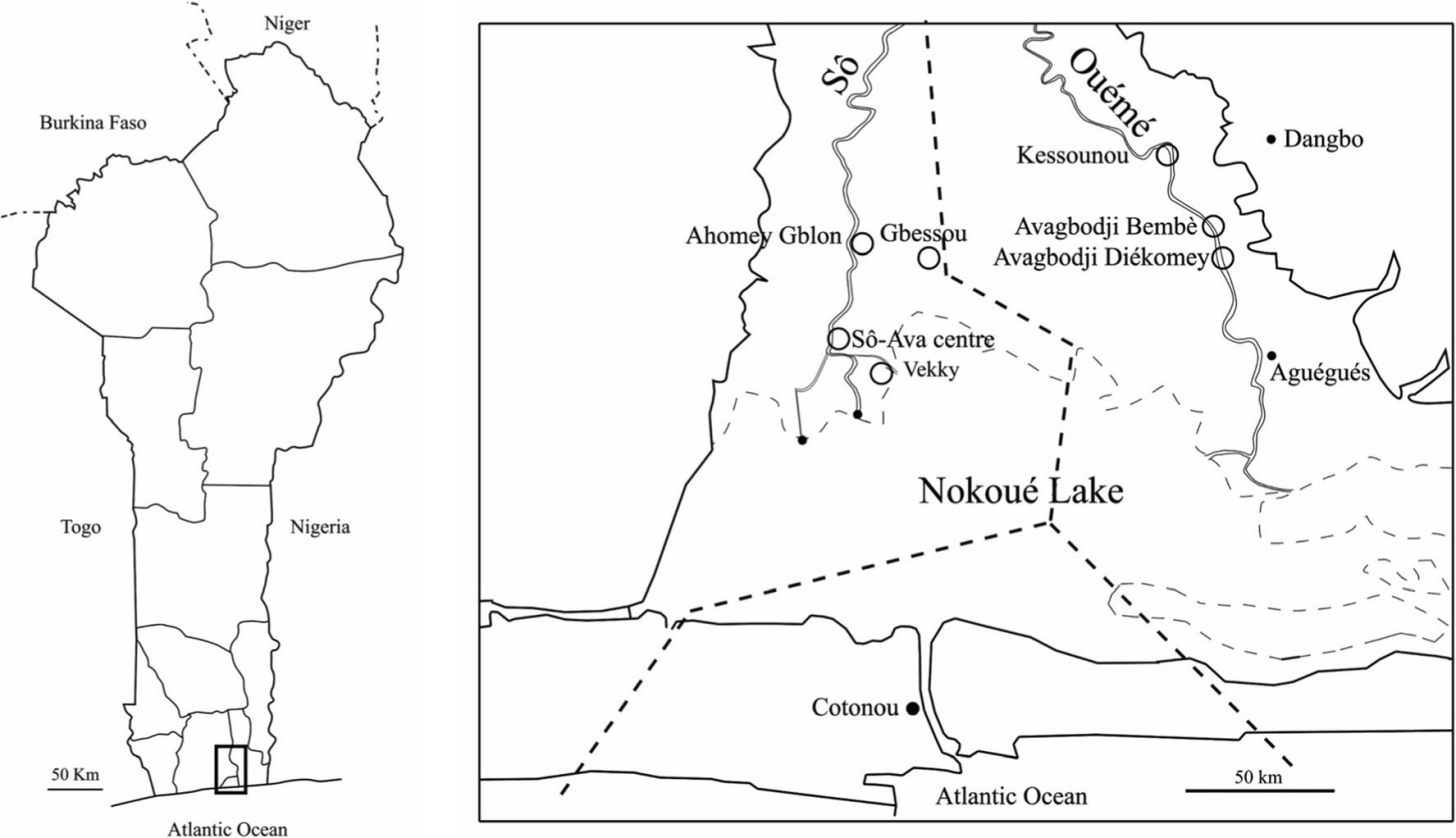
Geographical positions of the villages where samples were collected. **a** Position of the area in South Benin; **b** zoom view and position of the seven villages (white circles)

Small mammal sampling was performed in December 2019 in seven villages of southern Benin (Fig. 1): four villages within the Sô River hydrosystem (Ahomey Gblon, Gbessou, Sô-Ava Centre, and Vekky, all belonging to the Atlantic Department), and three along the Ouémé River (Kessounou, Avagbodji Bembè, and Avagbodji Diékomey, all belonging to the Ouémé Department). Two different areas of Kessounou were sampled: Kessounou Glo and Kessounou Bahoué located on the west and east banks of the Ouémé River, respectively.

### Rodent trapping and identification

Since trapping success of different rodent species may depend on the types of traps used (Garba et al. 2014), both Sherman traps (B. B. Sherman Traps, Inc., Tallahassee, FL, USA) and locally made wire-mesh live traps were systematically used together for a total of 498 night traps. Each trap was baited with the same mixture of peanut butter and fish and then set within houses over two successive nights. Captured animals were kept alive until their processing, which occurred within a week from the time of collection.

Rodents and shrews were identified using morphology, weight, and external measurements (head and body length, tail length, hindfoot length, and ear maximum length) according to standard taxonomy keys (Granjon and Duplantier 2009). However, coexistence of sibling species within African rodent genera is frequent. This is the reason why species-specific identification of all *Schistosoma*-carrying individuals was assessed following a complete cytochrome *b* mitochondrial gene sequencing as previously described in Dobigny et al. (2008).

### Adult schistosome recovery

**Fig. 2:**
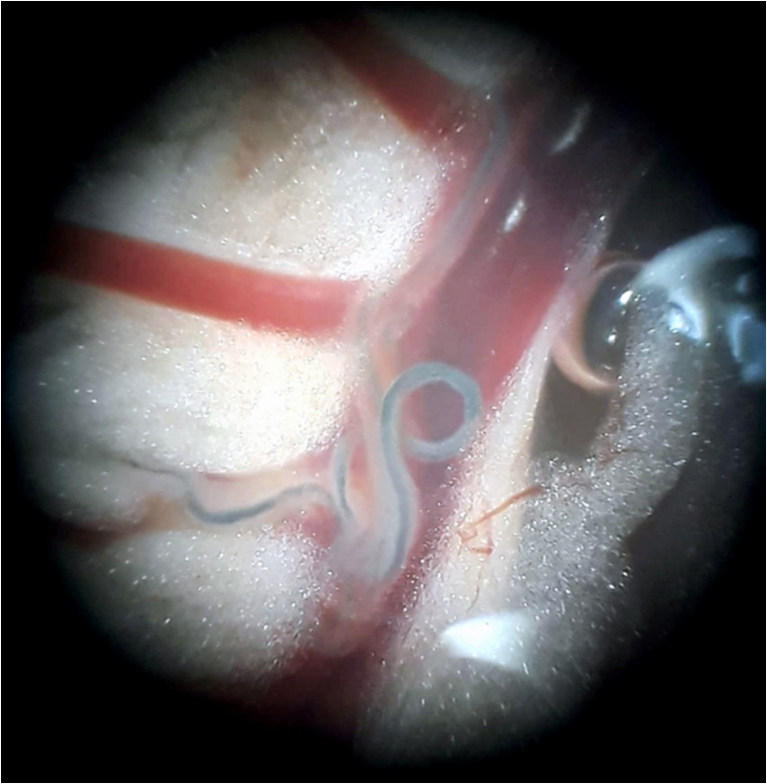
Adult paired worms in the mesenteric veins of *Mastomys natalensis*

The small mammals were euthanized by a lethal intraperitoneal injection of sodium pentobarbital. They were perfused (Duvall and DeWitt 1967) using Masterflex L/S apparatus (Cole-Parmer Instrument Co., Cambridgeshire, UK), and adult schistosomes were collected. The trapped worms in the liver and mesenteric (Fig. 2) or vesical veins were collected after excision of these organs under dissecting microscope. Male and female worms were numbered and placed in 95° alcohol for molecular analyses.

### *Schistosoma* egg morphology and miracidial hatching

Liver and duodenum of each rodent harboring *Schistosoma* adults were ground in saline solution (NaCl 0.9%) and filtered through a series of sieves (425-, 180-, 106-, and 45-μm, successively). The eggs were trapped on a 45-μm sieve. They were randomly sampled for both morphological and molecular studies and mounted individually in a 0.9% NaCl solution beneath glass cover slips on glass slides using a Pasteur pipette. They were then photographed and those which contained a living miracidium were measured (length, width, and spine length) under an optical microscope. In total, 24 schistosome eggs from *Mastomys natalensis* (Smith, 1834) and two eggs from *Rattus rattus* (Linnaeus, 1758) were measured*.* For molecular biology analyses, miracidial hatching was conducted by placing some eggs in a beaker containing well water and then several individual miracidia were plotted onto FTA® classic cards (Whatman, GE Healthcare companies, Little Chalfont, UK). The rest of the miracidia from the rodents were used for snail infections.

### Snail infection

Seventy-two *Bulinus globosus* (Morelet, 1866) (6.4 ± 1 mm height) were individually exposed to a single miracidium. The presence of only one miracidium was verified for each snail. This was made because we wanted that all the cercariae coming from each monomiracidially infected snail had the same genotype (one miracidium gives rise, by asexual multiplication, to thousands of cercariae of only one genotype). This ensured us that all the cercariae that were counted for the cercarial emission pattern analyses were from the same genotype and that multi-peaks were coming without any doubt from only one miracidium. The snails were then fed with dry and fresh lettuce and maintained individually in a glass containing 40 ml of well water at 26 °C with a balanced photoperiod (12-h light, 12-h dark) and a photophase from 6:00 am to 6:00 pm. Once a day, each snail was transferred to a new glass containing well water, and the cercarial emission was checked.

### Cercarial emergence pattern

The cercarial emergence pattern was analyzed every hour. For this analysis, each infected snail was transferred exactly every hour to a new glass containing 40 ml of well water at 26 °C; each transfer lasted approximately 3 s per snail. Regarding the specific transfers at 6 am and 6 pm, snails were transferred 1 min before light shifts. The water samples containing the emerged cercariae were filtered through polyamide nitrile filters (25-μm mesh sieve). The cercariae retained on the filter were stained with Lugol’s solution and counted under a dissecting microscope.

The cercarial emission pattern was analyzed every hour over 6 consecutive days, i.e., over a period of 144 h (6 × 24). Several fresh cercariae were individually stored onto FTA® classic cards for further molecular investigations.

### *Schistosoma* miracidium recoveries from urine and stool of schoolchildren

In order to get comparable data from the same period (December 2019) on schistosomiasis in humans, urine samples from 50 primary schoolchildren volunteers of Kessounou village (11 girls and 16 boys in the 4th grade; and 9 girls and 14 boys in the 5th grade) were collected individually and then mixed and passed through a 45-μm pore size sieve. The retained eggs were placed in a beaker containing well water for miracidial hatching. The stools from 46 of the primary schoolchildren were collected individually then mixed in a 0.9% NaCl saline solution. The mixture was passed through a series of sieves (425-, 180-, 106-, and 45-μm pore size, successively) and washed through with another 0.9% NaCl saline solution using a pressure pump. The eggs that were retained on the 45-μm pore size sieve were collected in a beaker containing well water for miracidial hatching. Using such a protocol, the infection status of each individual urine and feces sample could not be analyzed but all the schoolchildren were treated. Several miracidia retrieved from human urine and stool samples were collected individually and placed onto FTA® classic cards for further molecular analyses following the protocols detailed above.

### Molecular analyses

#### DNA extraction

Total DNA was extracted from each adult worm using the E.Z.N.A.® Tissue DNA Kit (Omega Bio-Tek, Norcross, GA, USA), as recommended by the manufacturer, and stored at −20 °C. DNA from miracidia and cercariae that were individually stored on FTA® classic cards were extracted as follows. 3-mm^2^ disks were cut and removed with a Craft Punch from the FTA® classic cards where the miracidium or cercaria was loaded. The disks were then deposited in Eppendorf tubes (1.5 ml) and an initial washing step with 100 μl of Milli-Q water was performed. After a 10-min incubation at room temperature, water was removed and replaced by 80 μl of 5% Chelex® 100 Molecular Biology Grade Resin solution (Bio-Rad Laboratories, Hercules, CA, USA). The samples were then heated to 65 °C for 30 min at a stirring speed of 800 rpm, followed by a second heating phase at 99 °C for 8 min without stirring. DNA was finally collected by centrifugation at 14,000 rpm for 2 min and 50 μl of the supernatant (DNA) of each sample were stored at −20 °C for further molecular analyses.

#### Rapid diagnostic multiplex polymerase chain reaction

A rapid diagnostic multiplex polymerase chain reaction (PCR) targeting the cytochrome C oxidase subunit I mitochondrial DNA gene (COI mtDNA) was conducted as described in Savassi et al. (2020). The primers used were one universal reverse primer (Shmb.R: 5′-CAA GTA TCA TGA AAY ART ATR TCT AA-3′) and three species-specific forward primers: Sh.F: 5′-GGT CTC GTG TAT GAG ATC CTA TAG TTT G-3′ for *S*. *haematobium* (120 bp); Sb.F: 5′-GTT TAG GTA GTG TAG TTT GGG CTC AC-3′ for *S*. *bovis* (260 bp); and Sm.F: 5′-CTT TGA TTC GTT AAC TGG AGT G -3′ for *S*. *mansoni* (215 bp).

#### COI mtDNA PCR

Partial COI mtDNA amplification was performed by PCR using the Cox1_schist F forward primer (5′- TCTTTRGATCATAAGCG-3′), and the Cox1_schist-R reverse primer (5′-TAATGCATMGGAAAAAAACA-3′) (Lockyer et al. 2003). PCR conditions used were similar to those described in Moné et al. (2015).

#### Internal transcribed spacer partial 18S, internal transcribed spacer 1, 5.8S, and internal transcribed spacer 2 ribosomal DNA PCR

PCR amplification of internal transcribed spacer (ITS) rDNA (partial 18S, internal transcribed spacer 1 (ITS1), 5.8S, and internal transcribed spacer 2 (ITS2)) was performed using the primers of Barber et al. (2000) (ITS4F: 5′-TCCTCCGCTTATTGATATGC-3′, and ITS5R: 5′-GGAAGTAAAAGTCGTAACAAGG-3′) as well as PCR conditions described in Savassi et al. (2020).

#### Sequencing

Partial COI mtDNA gene and ITS rDNA region were sequenced (Genoscreen; Lille, France) using the reverse primers; some regions were also sequenced using the forward primer in order to confirm the sequence. Sequences were all manually edited and verified using Sequencher 4.5 (Gene Codes Corporation, Ann Arbor, USA). The nucleotidic polymorphism of the nuclear sequences (ITS rDNA) was systematically investigated through the visualization of the raw chromatograms: since this gene has biparental transmission and segregation sites (polymorphic sites) between *S*. *haematobium* and *S*. *bovis*, the sequence chromatograms were carefully checked to identify the presence of possible heterozygous sites. At each polymorphic site (i.e., where two chromatogram peaks unambiguously overlapped, thus indicating the genetic signatures of both parent species), the IUPAC ambiguity symbols were used to indicate the individual nucleotide polymorphisms: thus, Y indicates the observed presence of both bases T and C, and not an ambiguous reading between T and C. Similarly, R indicates the observed presence of both bases A and G while W corresponds to the observed presence of both bases A and T.

#### Partial COI mtDNA phylogenetic analysis

DNA multiple sequence alignments were performed using Muscle program (Edgar 2004) implemented in MEGA 7.0 software (Kumar et al. 2016), and then refined using Gblocks 0.91b (Castresana 2000; Dereeper et al. 2008, 2010). The probabilistic model of sequence evolution (Nei and Kumar 2000) and the gamma distribution (*G*) to approximate rate heterogeneity among haplotypes was applied using MEGA 7.0 software. The best model showing the lowest BIC score (Bayesian information criterion) was HKY + G (0.16) (Hasegawa-Kishino-Yano with the gamma distribution). A phylogenetic tree using the maximum likelihood method was constructed using this model of molecular evolution under MEGA 7.0 software. Robustness of internal nodes was systematically assessed using bootstrapping procedures (1000 replicates). The topology of the tree was rooted using *S*. *intercalatum* Fisher, 1934 as a closely related but external outgroup.

### Statistical analyses

Means and standard errors were calculated. The Mann-Whitney test was used for morphological measure comparisons, and the Fisher’s exact test was implemented for the comparisons of proportions using BiostaTGV (https://biostatgv.sentiweb.fr). A *p* value less than 0.05 (<0.05) was considered to be statistically significant.

## Results

### Trapping success and schistosome prevalence in small mammals

In total, 62 small mammals were captured: 13/195 (6.7%) in Sherman traps and 49/303 (16.2%) in locally made wire-mesh traps (Fisher Exact Test, *p*=0.0031). The global trapping success was 12.5%, with local success ranging from 1.8% (Avagbodji Bembè) to 21.1% (Sô-Ava Centre) (Table 1). The small mammals included 61 rodents and 1 shrew (Table 1): 52 *Rattus rattus*, 3 *Rattus norvegicus* (Berkenhout, 1769), 6 *Mastomys natalensis*, and 1 *Crocidura* cf. *olivieri* (Lesson, 1827).

**Table 1:** Visited sites, micro-mammals trapped (*Crocidura olivieri*, *Mastomys natalensis* (Mn), *Rattus norvegicus*, *Rattus rattus* (Rr)), and infection with schistosomes

Area	Site	Trapping success	Perfused	Species	Sex	GPS coordinates		Schistosome infection status (code)
						Latitude	Longitude	
Sô-Ava	Ahomey Gblon	3/76 (4.0%)	3/3 *R. rattus*	*R. rattus*	Female	6.53763	2.40441	Negative
						6.53804	2.40452	
					Male	6.53810	2.40454	
	Gbessou	7/45 (15.6%)	4/5 *R. rattus* 0/1 *M. natalensis* 1/1 *C. olivieri*	*R. rattus*	Female	6.53096	2.43257	Negative
						6.53478	2.43913	
						6.53489	2.43885	
					Male	6.53559	2.43841	
				*C. olivieri*	Female	6.53473	2.43921	
	Sô-Ava Centre	15/71 (21.1%)	14/15 *R. rattus*	*R. rattus*	Female	6.49780	2.39962	Negative
						6.49782	2.39956	
						6.49792	2.39955	
						6.49794	2.39975	
						6.49796	2.39950	
						6.49820	2.39963	
						6.49833	2.39957	
						6.49835	2.39978	
						6.49870	2.39967	
						6.49911	2.40090	
						6.49939	2.40061	
						6.49939	2.40088	
					Male	6.49791	2.39978	
						6.49912	2.40093	
	Vekky	11/55 (20%)	5/8 *R. rattus* 3/3 *R. norvegicus*	*R. rattus*	Male	6.48694	2.42053	Positive (V42_Rr)
						6.48697	2.42075	Positive (V20_Rr)
						6.48744	2.42041	Positive (V132_Rr)
						6.48704	2.42075	Positive (V17_Rr)
						6.48704	2.42075	Negative
				*R. norvegicus*	Female	6.48704	2.42073	
						6.48746	2.42055	
						6.48753	2.42101	
	Total	36/247 (14.6%)	26/31 *R. rattus* 3/3 *R. norvegicus* 0/1 *M. natalensis* 1/1 *C. olivieri*					4 *R. rattus* positive
Dangbo	Kessounou Glo	9/64 (14.1%)	4/5 *R. rattus* 4/4 *M. natalensis*	*R. rattus*	Female	6.57703	2.52147	Negative
						6.57736	2.52158	
					Male	6.57730	2.52152	
						6.57738	2.52128	
				*M. natalensis*	Female	6.57735	2.52153	Positive (K14_Mn1)
						6.57730	2.52148	Positive (K14_Mn2)
					Male	6.57723	2.52151	Negative
						6.57724	2.52134	
	Kessounou Bahoué	11/76 (14.5%)	7/11 *R. rattus*	*R. rattus*	Female	6.57683	2.52259	Negative
						6.57684	2.52265	
						6.57685	2.52293	
						6.57690	2.52292	
					Male	6.57683	2.52296	
						6.57684	2.52275	
						6.57692	2.52275	
	Total	20/140 (14.3)	11/16 *R. rattus* 4/4 *M. natalensis*					2 *M. natalensis* positive
Aguégué	Avagbodji Bembè	1/55 (1.8%)	1/1 *R. rattus*	*R. rattus*	Female	6.53687	2.53622	Negative
	Avagbodji Diékomey	5/56 (8.9%)	3/4 *R. rattus* 1/1 *M. natalensis*	*R. rattus*	Female	6.52598	2.53695	Negative
				*R. rattus*	Female	6.52615	2.53701	
				*R. rattus*	Male	6.52601	2.53691	
				*M. natalensis*	Female	6.52622	2.53711	
	Total	6/111 (5.4%)	4/5 *R. rattus* 1/1 *M. natalensis*					Negative
Total		62/498 (12.5%)	41/52 *R. rattus*					4 positive
			3/3 *R. norvegicus*					Negative
			5/6 *M. natalensis*					2 positive
			1/1 *C. olivieri*					Negative

Among the 62 animals trapped, 50 individuals could be perfused and investigated for *Schistosoma* adults and eggs: 37 *R. rattus* (22 females and 15 males); 3 *R. norvegicus* (all females); 5 *M. natalensis* (3 females and 2 males) and 1 *C. olivieri* (female). Details by locality and species are provided in Table 1. In total, six rodents were found carrying *Schistosoma* adults: four male *R. rattus* from Vekky (V42_Rr*_*GDOB2229, V20_Rr*_*GDOB2231, V132_Rr*_*GDOB2232, and V17_Rr*_*GDOB2233) and two female *M. natalensis* from Kessounou Glo (K14_Mn*_*GDOB2246 and K34_Mn*_*GDOB2248). When considering only the individuals that could be perfused, this makes a species-specific prevalence of 9.8% (4/41) and 40% (2/5) in *R. rattus* and *M. natalensis*, respectively. Local prevalence reached 80% (4/5) for *R. rattus* from Vekky, and 50% (2/4) for *M. natalensis* from Kessounou Glo (Table 1).

### Schistosome burden and egg presence

Schistosome burden (number of adult schistosomes recovered from the rodents) was variable between the rodents.

In the four positive *R. rattus* from Vekky, we found 4 worms (2 females and 2 males) and only two eggs in V42_Rr (Field code=GDOB2229), 8 male worms in V20_Rr (GDOB2231), 3 worms (2 females, and 1 male) in V132_Rr (GDOB2232) and 1 male worm in V17_Rr (GDOB2233). The two eggs were used for morphology and all the adult worms were used for molecular studies.

In the two *M. natalensis* from Kessounou Glo, we found 30 worms (16 males and 7 pairs) in K14_Mn (GDOB2246) and 3 males in K34_Mn (GDOB2248). All of the adult worms were used for molecular studies. Several eggs could be extracted from K14_Mn and were used for both morphology and molecular studies.

### *Schistosoma* egg morphology

Two morphotypes were observed in the eggs isolated from *M. natalensis*: *S*. *haematobium* morphotype and an intermediate morphotype between *S*. *haematobium* and *S*. *bovis*. The percentages of each morphotype were not significantly different (41.7 and 58.3%, respectively; Fisher exact test, *p*>0.05). Regarding egg morphometry, mean egg length was significantly lower in the *S*. *haematobium* morphotype (164.00 μm±3.89; *N*=10) compared to the intermediate morphotype group (177.14 μm±3.88; *N*=14; Mann-Whitney test, *p*=0.029); the same trend was observed for the mean length/width ratio with 1.93±0.03 and 2.22±0.04, respectively (Mann-Whitney test, *p*=0.0001). The two eggs from *R. rattus* displayed intermediate morphotypes, the egg lengths were 165 and 240 μm and the mean length/width ratio 2.12 and 2.34, respectively.

### Snail infection

Among the 72 *B. globosus* that were exposed to one miracidium per snail, 52 survived and 3 shed cercariae (5.8%) (*Bg*1, *Bg*2, and *Bg*3) (the miracidia were all extracted from the *Mastomys natalensis* K14_Mn (GDOB2246) from Kessounou Glo). Prepatent periods lasted 39 days from *Bg*1, 45 days from *Bg*2, and 46 days from *Bg*3.

### Cercarial emission patterns

The cercarial emission patterns were analyzed for *Bg*1 and *Bg*2 over 6 consecutive days, from the 39th to the 44th day after infection for *Bg*1 and from the 49th to the 54th day after infection for the *Bg*2. The analysis could not be done for the *Bg*3 which died. The results showed that the two snails harbored different patterns.

**Fig. 3:**
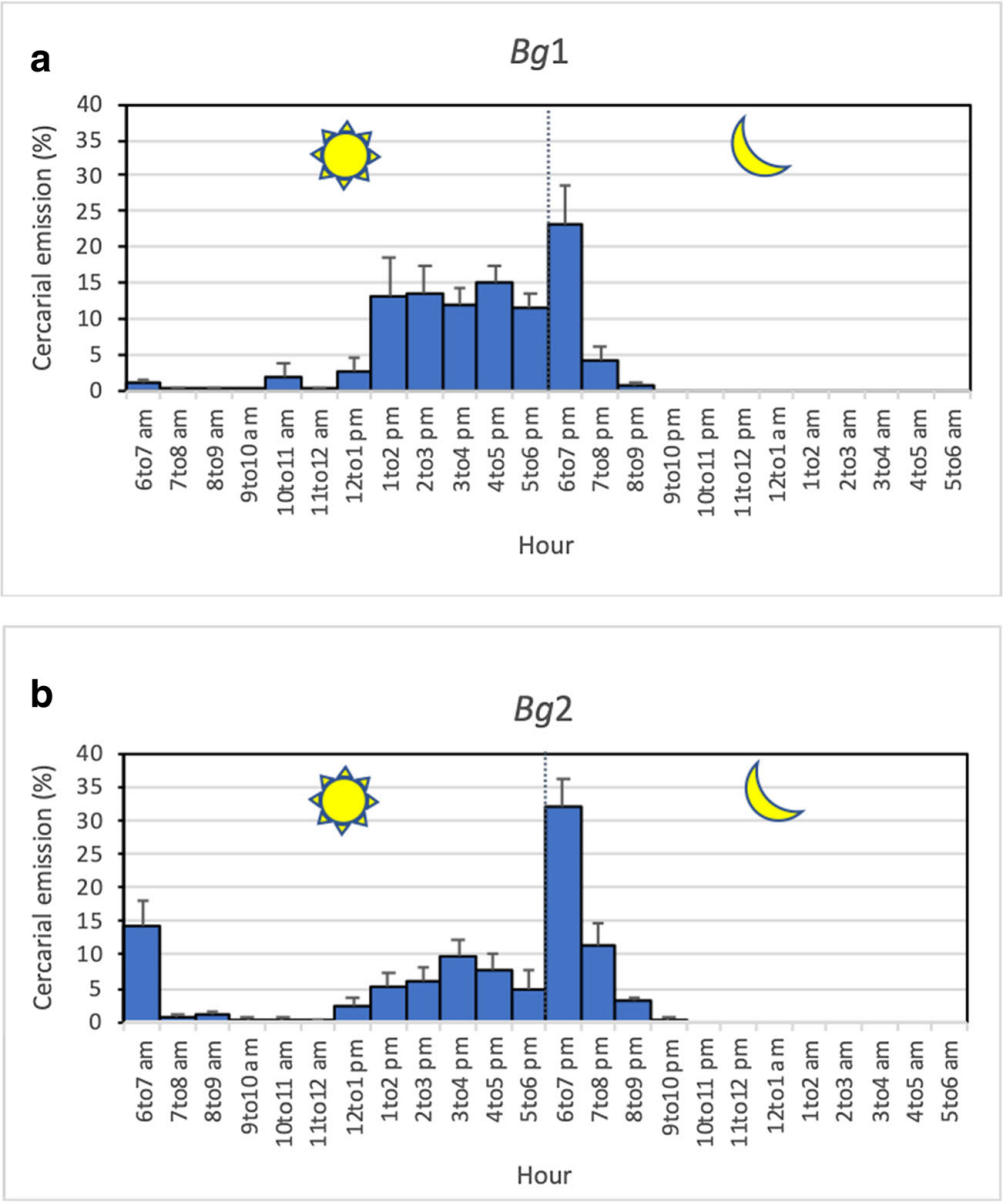
Cercarial emission patterns from infected snails, each exposed to 1 miracidium of *Schistosoma* from *Mastomys natalensis*. **a** Late diurnal and nocturnal pattern for *Bulinus globosus* (*Bg*1); **b** early and late diurnal and nocturnal pattern for *B. globosus* (*Bg*2)

The *Bg*1 snail had a late diurnal and nocturnal pattern, with 72% of the cercariae that were shed during the day. Cercarial emission occurred mainly from 2 to 7 pm, with a high plateau of emission between 1 and 6 pm and an emission peak focalized at 7 pm (Fig. 3a).

The *Bg*2 snail differed by the simultaneous presence of three peaks: an early diurnal peak at 7 am, a late diurnal peak at 4 pm, and a nocturnal peak at 7 pm. The percentage of cercariae shed during the day was nearly 53%. The nocturnal cercarial emission occurred as a very sharp peak at 7 pm, the first hour of darkness, representing 47% of the total cercarial daily emission (Fig. 3b).

### COI mtDNA (1027 bp)

One hundred and fifty-six sequences were analyzed and showed 30 mutation sites and 14 haplotypes (Table 2). From the 96 sequences of schistosomes extracted from the two *M. natalensis*, we found only 1 haplotype (K14M1_Mn_Hap1) shared by the 33 adult worms from the two *M. natalensis* (30 from K14_Mn and 3 from K34_Mn), by the 34 miracidia obtained from K14_Mn, and by the 29 cercariae emitted by the 3 snails infected with one miracidium (13, 14, and 2, respectively).

**Table 2:** The 14 haplotypes of COI mtDNA gene harbored by schistosomes from rodents and human

Host	Haplotype	30 polymorphic positions																														Schistosome stage harboring the haplotype (number of sequences)
		84	106	144	150	195	231	294	324	333	348	369	432	450	594	630	690	699	777	778	792	807	843	897	900	963	987	990	1008	1009	1026	
*M. natalensis* and *H. sapiens*	K14M1_Mn_Hap1	A	T	A	G	T	G	A	G	C	T	T	C	T	A	T	C	C	C	T	G	G	T	T	C	G	C	C	C	C	A	K14_Mn_Ad (30) K34_Mn_Ad (3) K14_Mn_Mi (34) K14_MnBg1_Ce (13) K14_MnBg2_Ce (14) K14_MnBg3_Ce (2)
																																KS_Hs_Mi (1)
*R. rattus*	V20M1_Rr_Hap2	**-**	**-**	**-**	**-**	**-**	**-**	**-**	**-**	**-**	**-**	**-**	**-**	**-**	G	**-**	**-**	T	T	**-**	**-**	**-**	**-**	**-**	**-**	A	A	**-**	**-**	**-**	**-**	V42_Rr_Ad (2) V20_Rr_Ad (4) V132_Rr_Ad (1)
	V20M2_Rr_Hap3	**-**	**-**	G	A	C	**-**	G	**-**	T	**-**	G	**-**	**-**	**-**	**-**	T	T	T	**-**	**-**	**-**	**-**	**-**	T	A	A	**-**	**-**	**-**	**-**	V20_Rr_Ad (1) V132_Rr_Ad (1)
	V20M3_Rr_Hap4	**-**	**-**	**-**	A	**-**	**-**	**-**	**-**	T	**-**	G	**-**	**-**	**-**	**-**	T	T	T	**-**	**-**	**-**	C	**-**	**-**	A	G	**-**	T	**-**	G	V42_Rr_Ad (2) V20_Rr_Ad (1)
	V132M1_Rr_Hap5	**-**	**-**	G	A	C	**-**	G	T	T	**-**	G	**-**	**-**	**-**	**-**	T	T	T	**-**	**-**	**-**	**-**	**-**	T	A	A	**-**	**-**	**-**	**-**	V132_Rr_Ad (1)
*H. sapiens*	KU1_Hs_Hap6	**-**	**-**	**-**	**-**	**-**	**-**	**-**	**-**	**-**	**-**	**-**	T	**-**	**-**	**-**	**-**	**-**	**-**	**-**	**-**	**-**	**-**	**-**	**-**	**-**	**-**	**-**	**-**	**-**	**-**	KU_Hs_Mi (11) KS_Hs_Mi (8)
	KU3_Hs_Hap7	**-**	**-**	**-**	**-**	**-**	**-**	**-**	**-**	**-**	**-**	**-**	T	**-**	**-**	**-**	**-**	**-**	**-**	**-**	A	**-**	**-**	**-**	**-**	**-**	**-**	**-**	**-**	**-**	**-**	KU_Hs_Mi (3) KS_Hs_Mi (2)
	KU4_Hs_Hap8	**-**	**-**	**-**	**-**	**-**	**-**	**-**	**-**	**-**	**-**	**-**	**-**	**-**	**-**	**-**	T	**-**	**-**	**-**	**-**	**-**	**-**	**-**	**-**	**-**	**-**	**-**	**-**	**-**	**-**	KU_Hs_Mi (3) KS_Hs_Mi (8)
	KU13_Hs_Hap9	**-**	C	**-**	**-**	**-**	**-**	**-**	**-**	**-**	**-**	**-**	**-**	**-**	**-**	**-**	**-**	**-**	**-**	**-**	**-**	**-**	**-**	**-**	**-**	**-**	**-**	**-**	**-**	**-**	**-**	KU_Hs_Mi (2) KS_Hs_Mi (1)
	KU5_Hs_Hap10	**-**	**-**	**-**	**-**	**-**	**-**	**-**	**-**	**-**	**-**	**-**	T	**-**	**-**	**-**	**-**	**-**	**-**	**-**	**-**	**-**	**-**	C	**-**	**-**	**-**	**-**	**-**	**-**	**-**	KU_Hs_Mi (1)
	KU10_Hs_Hap11	G	**-**	**-**	**-**	**-**	**-**	**-**	**-**	**-**	**-**	**-**	**-**	**-**	**-**	C	**-**	T	T	C	**-**	**-**	**-**	**-**	**-**	A	A	T	**-**	**-**	**-**	KU_Hs_Mi (2) KS_Hs_Mi (1)
	KU11_Hs_Hap12	G	**-**	**-**	**-**	**-**	**-**	**-**	**-**	**-**	C	**-**	**-**	**-**	**-**	C	**-**	T	T	**-**	**-**	A	**-**	**-**	**-**	A	A	T	**-**	**-**	**-**	KU_Hs_Mi (1)
	KS5_Hs_Hap13	**-**	**-**	**-**	**-**	**-**	**-**	**-**	**-**	**-**	**-**	**-**	T	C	**-**	**-**	**-**	**-**	**-**	**-**	**-**	**-**	**-**	**-**	**-**	**-**	**-**	**-**	**-**	**-**	**-**	KS_Hs_Mi (1)
	KS10_Hs_Hap14	**-**	**-**	**-**	**-**	**-**	A	**-**	**-**	**-**	**-**	**-**	T	**-**	**-**	**-**	**-**	**-**	**-**	**-**	**-**	**-**	**-**	**-**	**-**	**-**	**-**	**-**	**-**	**-**	**-**	KS_Hs_Mi (2)

K, Kessounou; V, Vekky; Bg, *Bulinus globosus*; Hs, *Homo sapiens*; Mn, *Mastomys natalensis*; Rr, *Rattus rattus*; Ad, adults; Ce, cercaria; Mi, miracidium; S, stool; U, urine; Hap, haplotype

Among the 13 sequences of schistosomes that were obtained from the four *R. rattus*, four haplotypes were identified: V20M1_Rr_Hap2, V20M2_Rr_Hap3, V20M3_Rr_Hap4, and V132M1_Rr_Hap5, all different from the haplotype K14M1_Mn_Hap1 found from *M. natalensis*. One to three of these *Rattus*-originating haplotypes were found within the same *R. rattus* individual.

From the 23 sequences of schistosomes retrieved from children’s urine, we found 7 haplotypes (KU1_Hs_Hap6; KU3_Hs_Hap7; KU4_Hs_Hap8; KU13_Hs_Hap9; KU5_Hs_Hap10; KU10_Hs_Hap11; KU11_Hs_Hap12) all different from those extracted from rodents. From the 24 sequences of schistosomes extracted from children stool, we found 8 haplotypes (KU1_Hs_Hap6; KU3_Hs_Hap7; KU4_Hs_Hap8; KU13_Hs_Hap9; KU10_Hs_Hap11, KS5_Hs_Hap13, KS10_Hs_Hap14), among which five were shared with some haplotypes found in the urine, and one haplotype (K14M1_Mn_Hap1) shared with the schistosome haplotypes found in *M. natalensis*. Sequence data were deposited in the NCBI GenBank database under the accession numbers from MW022134 to MW022147.

### COI mtDNA phylogeny (884 bp)

**Fig. 4:**
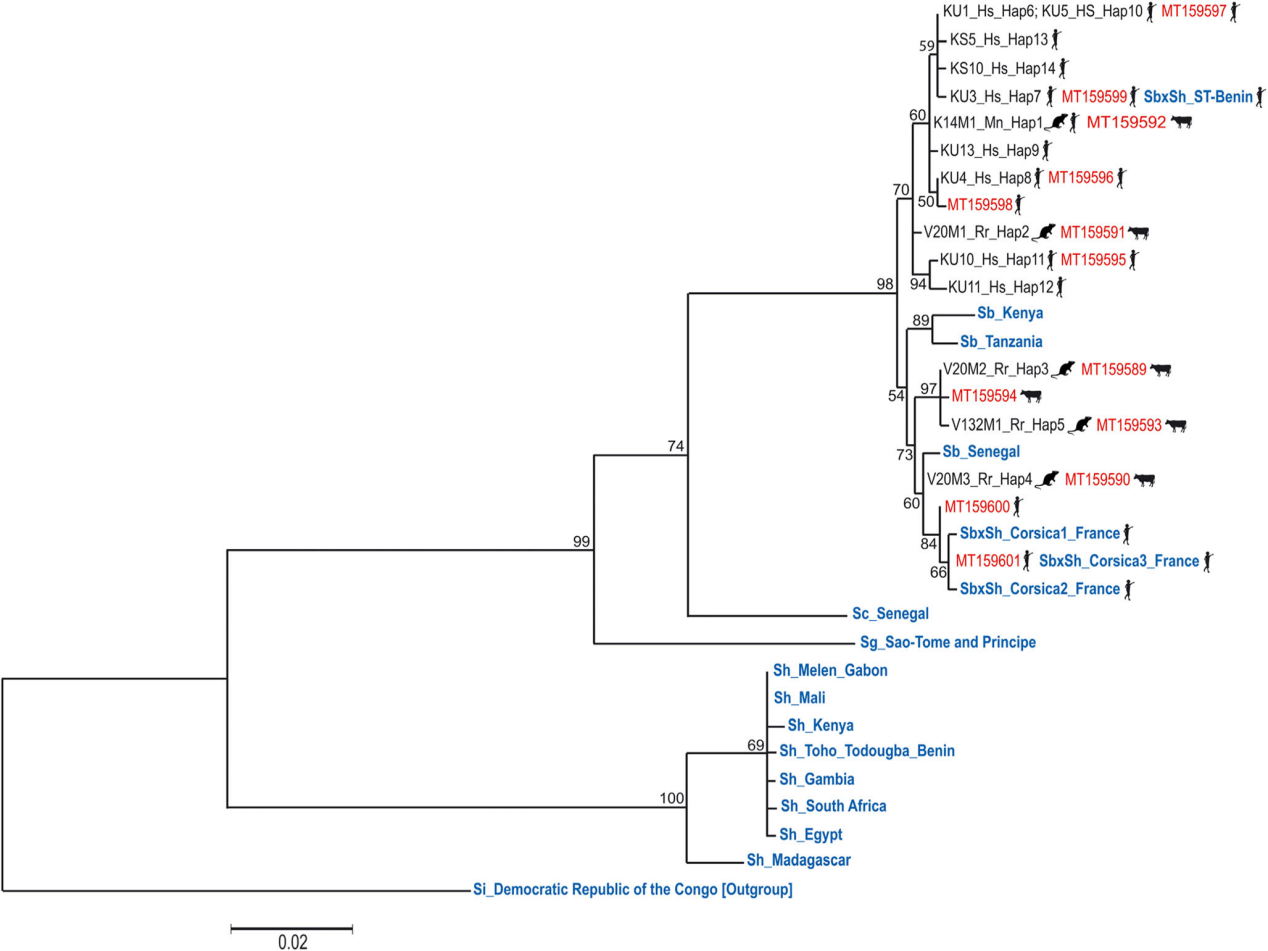
Maximum likelihood tree topology built with 14 haplotypes COI mtDNA (884 bp) showing that the schistosomes recovered from either rodents or schoolchildren (in black) belong to the *S. bovis* clade. The scale shows the number of nucleotide substitutions per site. Comparison was made with GenBank sequences (in red) obtained by Savassi et al. (2020) and sequences (in blue) obtained from various other references. Haplotype code/COI mtDNA accession nos.: K14M1_Mn_Hap1/MW022134; V20M1_Rr_Hap2/ MW022135; V20M2_Rr_Hap3/ MW022136; V20M3_Rr_Hap4/ MW022137; V132M1_Rr_Hap5/ MW022138; KU1_Hs_Hap6/MW022139; KU3_Hs_Hap7/MW022140; KU4_Hs_Hap8/MW022141; KU13_Hs_Hap9/MW022142; KU5_Hs_Hap10/MW022143; KU10_Hs_Hap11/MW022144; KU11_Hs_Hap12/MW022145; KS5_Hs_Hap13/MW022146; KS10_Hs_Hap14/MW022147

The 14 haplotypes obtained from the COI mtDNA sequences (MW022134 to MW022147) were compared to 18 nucleotide sequences of *Schistosoma* with terminal-spined eggs (Rollinson and Southgate 1987) available in GenBank: 8 sequences of *S*. *haematobium*, from Gambia (JQ397349), Mali (AY157209), Egypt (JQ397368), Toho-Todougba_Benin (KT354661), Melen_Gabon (KT354660), Kenya (JQ397378), South Africa (JQ397397), and Madagascar (JQ397399); 3 sequences of *S*. *bovis*, from Senegal (AJ519521), Kenya (FJ897160), and Tanzania (AY157212); 4 sequences of *S*. *bovis* x *S*. *haematobium*, from Corsica, France (KT354656, KT354657, KT354658), and Sô-Tchanhoué, Benin (KT354662); 1 sequence of *S*. *guineensis* Pagès, Jourdane, Southgate & Tchuem Tchenté, 2003 from Sao Tome and Principe (AJ519517); 1 sequence of *S*. *curassoni* Brumpt, 1931 from Senegal (AJ519516); and 1 sequence of *S*. *intercalatum* from Democratic Republic of the Congo (AJ519515). Maximum likelihood tree topology showed that all the 14 Beninese haplotypes, which came either from rodents or schoolchildren, belonged to the *S*. *bovis* and *S*. *bovis* x *S*. *haematobium* clade, but not to the *S*. *haematobium* or to other *Schistosoma* species clades (Fig. 4). Genetic differences ranged between 0.11 and 1.13% among the haplotypes obtained from rodents; they ranged between 0 and 1.36% when both rodent- and human-originating haplotypes were considered. The genetic difference between schistosome haplotypes obtained from Benin rodents and the *S*. *bovis* haplotypes from Senegal, Kenya, and Tanzania ranged between 0.23 and 1.47%; this difference ranged between 0.23 and 1.36% between the rodent-originating haplotypes and the previously published *S*. *bovis* x *S*. *haematobium* haplotypes from Benin and Corsica. The five haplotypes recovered from rodents were similar to haplotypes from cows, and one of them (K14M1_Mn_Hap1) was identical to a haplotype from humans.

### ITS rDNA (946 bp)

One hundred and sixty-one 18S, ITS1, 5.8S, and ITS2 sequences were analyzed. The first 22 bp of our ITS rDNA sequences belonged to the 18S gene and no variability was found between any of our samples. We thus no longer gave this gene further consideration. Eleven profiles were found with the ITS1, 5.8S, and ITS2 sequences, with 1 to 3 profiles per rodent, only 1 profile for all the miracidia, only one profile for all the cercariae, and 6 and 7 profiles for urine and stool of schoolchildren, respectively (Table 1). Sequence data were deposited in the NCBI GenBank database under the accession numbers from MW027648 to MW027658.

Table 3 shows that the 11 profiles differ by their ITS1 gene (458 bp: from position 23 to position 480), with 5 genetic variants at four positions (TGAT, TGAY, TAAT, TART, and YAAT); by their 5.8S gene (155 bp: from position 481 to position 635), with 2 genetic variants at 1 position (C and Y); and by their ITS2 gene (311 bp: from position 636 to position 946), with 5 genetic variants at 6 positions (AATGAT, WATRAT, AATRAT, AGCGGC, and ARYGRY). Among these 11 profiles, four were found only in *R. rattus* (profiles 1 to 4: accession numbers MW027648 to MW027651), one profile corresponded to the typical *S*. *bovis* ITS rDNA (profile 1: accession number MW027648) and three profiles showed variants of the *S*. *bovis* typical profile with double peaks either in the ITS1 or in the ITS2 gene (profiles 2 to 4: accession numbers MW027649 to MW027651). Two profiles were found in both *M. natalensis* and schoolchildren (profiles 5 and 6: accession numbers MW027652 and MW027653), with profile 5 harboring both a typical *S*. *haematobium* ITS1 gene together with a typical *S*. *bovis* ITS2 gene, and profile 6 harboring a new combination which included the ITS1 gene (TART, variant of *S*. *haematobium*) together with a typical *S*. *bovis* ITS2 gene. Profile 6 was observed in all the miracidia and all the cercariae collected from the *Bg*1, *Bg*2, and *Bg*3 snails experimentally infected with one miracidium extracted from naturally infected *M.* *natalensis*. The other five profiles were found only in schoolchildren (profiles 7 to 11: accession numbers MW027654 to MW027658) and showed variants of the *S*. *haematobium* typical profile, with double peaks in the chromatograms of at least one of the ITS1, 5.8S, or ITS2 genes.

**Table 3:** The 11 profiles of ITS rDNA region harbored by the schistosome from rodents and humans

Schistosome species	Profile	ITS1				5.8S	ITS2						Host	Schistosome stage harboring the profile (number of sequences)
		41	73	246	257	556	646	725	780	783	830	900		
*S. bovis* and its variants	1	T	G	A	T	C	A	A	T	G	A	T	*R. rattus*	V42_Rr_Ad (3) V20_Rr_Ad (5) V132_Rr_Ad (2) V17_Rr_Ad (1)
	2	T	G	A	Y	C	A	A	T	G	A	T		V42_Rr_Ad (1) V20_Rr_Ad (1)
	3	T	G	A	T	C	W	A	T	R	A	T		V132_Rr_Ad (1)
	4	T	G	A	T	C	A	A	T	R	A	T		V20_Rr_Ad (1)
*S. haematobium* x *S. bovis*	5	T	A	A	T	C	A	A	T	G	A	T	*M. natalensis* and *H. sapiens*	K14_Mn_Ad (15) KU_Hs_Mi (1) KS_Hs_Mi (2)
	6	T	A	R	T	C	A	A	T	G	A	T		K14_Mn_Ad (15) K34_Mn_Ad (3) K14_Mn_Mi (34) K14_MnBg1_Ce (13) K14_MnBg2_Ce (17) K14_MnBg3_Ce (2) KS_Hs_Mi (1)
	7	T	A	A	T	C	A	R	Y	G	R	Y	*H. sapiens*	KU_Hs_Mi (2) KS_Hs_Mi (1)
	8	T	A	A	T	Y	A	G	C	G	G	C		KU_Hs_Mi (7) KS_Hs_Mi (7)
	9	T	A	A	T	Y	A	R	Y	G	R	Y		KU_Hs_Mi (9) KS_Hs_Mi (8)
	10	Y	A	A	T	Y	A	G	C	G	G	C		KU_Hs_Mi (3) KS_Hs_Mi (4)
	11	Y	A	A	T	C	A	R	Y	G	R	Y		KU_Hs_Mi (1) KS_Hs_Mi (1)

Y indicates the presence of the bases T and C, rather than an ambiguous reading between T and C. Similarly, R indicates the presence of the bases A and G and not an ambiguous reading between A and G, and W indicates the presence of the bases A and T and not an ambiguous reading between A and T. K, Kessounou; V, Vekky; Bg, *Bulinus globosus*; Hs, *Homo sapiens*; Mn, *Mastomys natalensis*; Rr, *Rattus rattus*; Ad, adults; Ce, cercaria; Mi, miracidium; S, stool; U, urine

## Discussion

The rodent species assemblage identified here in the commensal compartment of the Lake Nokoué ecosystem is quite similar to those observed in the urban and peri-urban areas of south Benin, including the lacustrine city of Ganvié (Hima et al. 2019; Houéménou et al. 2019). Such a small mammal community has already been associated with the circulation of various zoonotic agents in this area: *Bartonella* (Leulmi et al. 2014; Martin-Alonso et al. 2016), water-associated *Leptospira* (Houéménou et al. 2019), *Rickettsia* (Leulmi et al. 2014), and *Trypanosoma* (Dobigny et al. 2019). The results obtained in the present manuscript showed that two species of rodents were found to harbor schistosomes: *Rattus rattus* and *Mastomys natalensis*. Although sampling size was quite low, the schistosome host-specific prevalence observed here was quite high: 80% in *R. rattus* from Vekky and 50% in *M. natalensis* from Kessounou Glo. For *R. rattus*, the four COI schistosome haplotypes and the four ITS profiles belong to *S*. *bovis* species. To our knowledge, this is the first time that *R. rattus* has been found to be naturally infected by *S*. *bovis*. This is surprising for two reasons: (i) *S*. *bovis* is known to have a large vertebrate definitive host spectrum, including rodent species, but not *R. rattus* (*Mastomys natalensis* and *Lophuromys flavopunctatus* Thomas 1888 by Pitchford (1977) and by Hanelt et al. (2010); *Arvicanthis niloticus* (Desmarest, 1822) by Catalano et al. (2018)); (ii) *R. rattus* is widespread in the lacustrine and semi-lacustrine habitats of south Benin (Hima et al. 2019; Houéménou et al. 2019) and is known to act as a natural host for other species of schistosomes such as *S*. *japonicum* in the Philippines (Fedorko, 1999) and *S*. *mansoni* in the West Indies (Combes et al. 1975) and in Oman (Mouahid et al. 2012). Limited by the fact that only two dead eggs were found, we were unable to prove that *R. rattus* plays an effective role in the transmission of *S*. *bovis*. Further work should be done on the role of *R. rattus* in the transmission of schistosomes in the researched zone. For *M. natalensis*, the molecular analysis of the different stages of the collected schistosomes showed a COI of the *S*. *bovis* type and an ITS of the *S*. *haematobium* x *S*. *bovis* type. Previously, in Senegal, Catalano et al. (2018) found one schistosome pair composed of a *S*. *mansoni* male with a hybrid female presenting the profile of *S*. *haematobium* for ITS and *S*. *bovis* for COI, in *Mastomys huberti* Wroughton, 1909; however, they did not provide any evidence of the presence of eggs or miracidia that could infect snails and shed cercariae. Our results make thus *M. natalensis* a natural host of an introgressive hybrid population between *S*. *haematobium* and *S*. *bovis* in the studied area. We provide the first example of a rodent being involved in the transmission of introgressive hybrid populations between *S*. *haematobium* and *S*. *bovis.* Indeed, the miracidia of these introgressive hybrids succeeded to infect snails and to give rise to cercariae. Such introgressive hybrid populations were also found in humans (this paper) and in bovines (Savassi et al. 2020) in the same geographical area; in humans, neither pure *S*. *haematobium* nor *S*. *bovis* species were found, while in bovines, *S*. *bovis* was present. Together, the three hosts, humans, rodents, and bovines, were found to share the same habitat as well as an introgressive hybrid schistosome population. The absence of pure *S*. *haematobium* may be surprising; however, the intensity of the interactions between the two species, *S*. *haematobium* and *S*. *bovis*, is very high, particularly in the studied area where bovines, humans, and rodents are living with high proximity (we collected the rodents in the human stilt houses) and can explain in this area that all the schistosomes that we collected were introgressive hybrids between the two species. Nothing excludes the effective presence of *S*. *haematobium* in other parts of Benin but this has to be investigated.

**Fig. 5:**
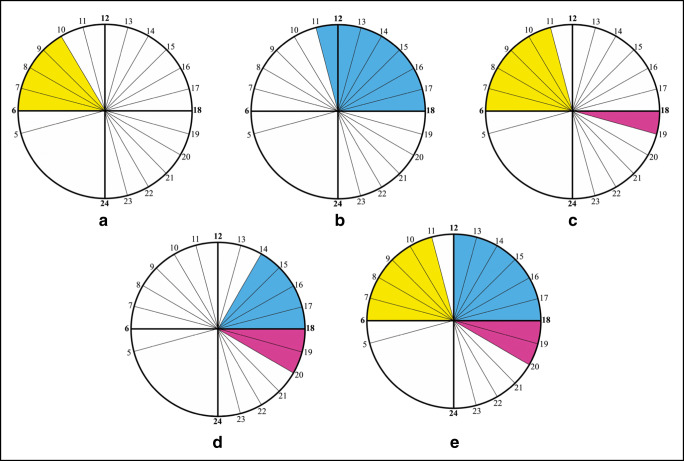
The study of schistosomiasis transmission at Kessounou (Benin), based on the present paper and on Savassi et al. (2020) data, showed five distinct cercarial emission patterns. Three populations of cercariae are clearly present: in yellow those which emerge early in the morning, corresponding to *S. bovis* profile, in blue those which emerge in the afternoon, corresponding to *S. haematobium* profile, and in pink those that emerge at nightfall, corresponding to the involvement of *Mastomys natalensis* (nocturnal rodent), in the transmission. The *bovis* profile (**a**) and *haematobium* profile (**b**) appear alone (one peak per day) or each associated with the nocturnal profile (two peaks per day) (**c**, **d**). The three populations can be associated with three peaks per day (**e**)

Two different *Mastomys*-related chronotypes were found and came from two different snails, each infected with a single miracidium. The first chronotype, obtained from the *Bg*1 snail, exhibited a late diurnal and nocturnal pattern (Fig. 3a). We recently identified a similar pattern for schistosomes collected from cows in the very same area (Savassi et al. 2020): it was the first time that a nocturnal shedding pattern was observed for schistosomes from cattle and led us to question the role of rodents as nocturnal animals. The second chronotype, obtained from the *Bg*2 snail (Fig. 3b), corresponds to a new chronotype with three peaks: an early diurnal, a late diurnal, and a nocturnal pattern. This chronotype cumulates all of the time periods associated with cercarial shedding patterns known to date: *S*. *bovis* typical pattern (early morning), *S*. *haematobium* typical pattern (diurnal or late diurnal), and a yet undescribed nocturnal pattern within *S*. *haematobium* and *S*. *bovis* species. The nocturnal period of cercarial emergence observed in both chronotypes can be linked to the nocturnal aquatic activity of *M. natalensis*. Duplantier and Granjon (1990) showed via experimentation that the activity pattern of *M. natalensis* is primarily nocturnal. *M. natalensis* is the most widespread and common rodent in Africa south of the Sahara; it has a semicommensal to commensal habitat, where it plays primordial role in the spread of zoonotic diseases and is dependent on the presence of water (Coetzee 1975; Christensen 1996; Granjon and Duplantier 2009). Nocturnal cercarial emergences have already been observed in other species of schistosomes, namely, *S*. *rodhaini* (Théron 1989), *S*. *mansoni* (Mouahid et al. 2012), and *S*. *japonicum* (Su et al. 2013), and were all related to the role of rodent hosts in the transmission. The fact that both of the *Mastomys*-derived chronotypes, the one with both late diurnal and nocturnal patterns and the one with the three peaks (early diurnal, late diurnal, and nocturnal), came from snails infected with only one miracidium puts into question the circadian nature of all of the previously published results on schistosome chronobiology. The situation in which the same cercariae “clone” (since issued from only one miracidium) exhibits distinct periods of cercarial emission during the same day was demonstrated for the first generation of offspring, during laboratory crossbreeding experiments, between two *S*. *mansoni* chronotypes with two different cercarial emission patterns: early diurnal and late diurnal (Théron and Combes 1983). These authors hypothesized genetic support for the cercarial emission with genetic codominance (the two phenotypes are expressed). More data are now needed regarding the frequency of the *M. natalensis*–derived chronotypes observed in the studied area and on their genetic and transcriptomic determinants. With the specific mechanisms still unknown, we can still propose a scenario for the various events which led to the establishment of the various chronotypes described in Kessounou (Benin), including those of Savassi et al. (2020). Five chronotypes were identified (Fig. 5): one-peak *S*. *bovis* typical chronotype (Fig. 5a), one-peak *S*. *haematobium* typical chronotype (Fig. 5b), two-peak *S*. *bovis* and nocturnal chronotype (Fig. 5c), two-peak *S*. *haematobium* and nocturnal chronotype (Fig. 5d), and three-peak *S*. *bovis*, *S*. *haematobium*, and nocturnal chronotype (Fig. 5e). It can be noted that two other possible chronotypes are absent: the chronotype with the nocturnal emission alone and the two-peak *S*. *bovis* and *S*. *haematobium* chronotype. Molecular analysis based on two genes (COI and ITS) has shown that the species *S*. *bovis* is only present in cattle, the species *S*. *haematobium* is absent, and the introgressive hybrid *S*. *haematobium* x *S*. *bovis* is present in cattle, schoolchildren, and *M. natalensis* (this paper and Savassi et al. 2020). The species *S*. *haematobium* seems to give way to its descendants only through introgressive hybridization with *S*. *bovis*, descendants which seem adapted to the three hosts. However, it can be noted that the cercarial emission pattern of the *S*. *haematobium* is maintained in the introgressive hybrid descendants (see Fig. 5b). Concerning the time position of the different peaks, the morning peak is comparable to a typical *S*. *bovis* peak (Mouahid and Théron 1986; Mouahid et al. 1987, 1991); in this case, the cercariae are shed when cattle go to watering. The afternoon peak is comparable to a typical *S*. *haematobium* peak (Mouahid et al. 1991); in this case, the cercariae are shed when humans go to water for bathing, washing, or fishing. The nocturnal peak is comparable to a typical *S*. *rodhaini* Brumpt 1931 peak and to the nocturnal chronotype of *S*. *mansoni* (Théron 1989; Mouahid et al. 2012); in this case, the cercariae are shed when nocturnal rodents actively use water.

In conclusion, our results showed for the first time that a rodent, precisely *Mastomys natalensis*, can act as a definitive host for transmission of introgressive hybrids between *S*. *haematobium* and *S*. *bovis*. This result was obtained using different complementary approaches: egg morphology, cercarial emergence patterns, and molecular biology. The morphological parameter did not constitute a precise tool for diagnose but was useful to alert us on the presence of unusual schistosome egg morphology in rodents since around 50% of eggs were typical *S. haematobium* eggs and the other 50% were of intermediate morphotype between *S. haematobium* and *S. bovis* eggs. The cercarial emission pattern, even though obtained from only two snails infected with one miracidium of *M. natalensis*–derived schistosomes, showed that the typical cercarial emergence patterns of *S. haematobium* and *S. bovis* could be found but associated with other periods of emergence, notably a nocturnal one; this abled us to make the link with the previous nocturnal cercarial shedding that we could observe in bovines (Savassi et al. 2020). Finally, the molecular parameter, based on both mitochondrial and nuclear genes, showed that those *M. natalensis–*derived schistosomes were introgressive hybrids between *S. haematobium* and *S. bovis*. More generally, schistosomes’ adaptive strategy towards their different hosts aims to optimize host ecological niche, host spectrum, and cercarial emission time period. In Benin, intra-host ecological niche is optimally used by the *S. haematobium* x *S. bovis* individuals since, in children, both the urine and feces are used for parasite transmission. The adaptive use of a multiple-host spectrum also seems optimized since humans as well as *Mastomys* (this manuscript) and bovines (Savassi et al. 2020) share the same genetic variants (see Fig. 4). Finally, we showed that parasite time period transmission within a single day is also maximized since daily (morning to late afternoon) and nocturnal cercarial shedding is observed for the same single schistosome genotype.

## Data Availability

Sequence data were deposited in the NCBI GenBank database under the accession numbers MW022134 to MW022147 for COI mtDNA and MW027648 to MW027658 for ITS rDNA.

## References

[CR1] Barber K, Mkoji G, Loker E (2000). PCR-RFLP analysis of the ITS2 region to identify *Schistosoma haematobium* and *S. bovis* from Kenya. Am J Trop Med Hyg.

[CR2] Boissier J, Mouahid G, Moné H (2019) *Schistosoma* spp. *In*: J.B. Rose and B. Jiménez-Cisneros, (eds) Global water pathogen project. http://www.waterpathogens.org (http://www.waterpathogens.org/) (Robertson, L (eds) Part 4 Helminths) http://www.waterpathogens.org/book/schistosoma (https://www.waterpathogens.org/book/schistosoma) Michigan State University, E. Lansing, MI, UNESCO.

[CR3] Castresana J (2000). Selection of conserved blocks from multiple alignments for their use in phylogenetic analysis. Mol Biol Evol.

[CR4] Catalano S, Sène M, Diouf ND, Fall CB, Borlase A, Léger E, Bâ K, Webster JP (2018). Rodents as natural hosts of zoonotic *Schistosoma* species and hybrids: an epidemiological and evolutionary perspective from West Africa. J Infect Dis.

[CR5] Christensen JT (1996). Home range and abundance of *Mastomys natalensis* (Smith, 1834) in habitats affected by cultivation. Afr J Ecol.

[CR6] Coetzee CG (1975). The biology, behaviour, and ecology of *Mastomys natalensis* in southern Africa. Bull World Health Organ.

[CR7] Combes C, Léger N, Golvan YJ (1975) Le rôle du Rat dans la dynamique de l’endémie schistosomienne en Guadeloupe. C R Acad Sci Paris 281, Série D, 1059–1061 813876

[CR8] Combes C, Fournier A, Moné H, Théron A (1994). Behaviors in trematode cercariae that enhance parasite transmission: patterns and processes. Parasitology.

[CR9] Dereeper A, Guignon V, Blanc G, Audic S, Buffet S, Chevenet F, Dufayard JF, Guindon S, Lefort V, Lescot M, Claverie JM, Gascuel O (2008) Phylogeny.fr: robust phylogenetic analysis for the non-specialist. Nucl Acids Res 36 (Web Server issue):W465–W469. 10.1093/nar/gkn180 10.1093/nar/gkn180 PMC2447785 18424797

[CR10] Dereeper A, Audic S, Claverie JM, Blanc G (2010). BLAST-EXPLORER helps you building datasets for phylogenetic analysis. BMC Evol Biol.

[CR11] Dobigny G, Lecompte E, Tatard C, Gauthier P, Bâ K, Denys C, Duplantier JM, Granjon L (2008). An update on the taxonomy and geographic distribution of the cryptic species *Mastomys kollmannspergeri* (Muridae, Murinae) using combined cytogenetic and molecular data. J Zool.

[CR12] Dobigny G, Gauthier P, Houéménou G, Dossou HJ, Badou S, Etougbétché J, Tatard C, Truc P (2019) Spatio-temporal survey of small mammal-borne *Trypanosoma lewisi* in Cotonou, Benin, and the potential risk of human infection. Infect Genet Evol 75: art. no [103967] 10.1016/j.meegid.2019.103967 31344489

[CR13] Duplantier JM, Granjon L (1990) Rythmes d’activité chez six espèces de Muridés du Sénégal appartenant aux genres *Mastomys*, *Arvicanthis*. *Myomys* et *Dasymys* Mammalia:173–182

[CR14] Duvall RH, DeWitt WB (1967). An improved perfusion technique for recovering adult schistosomes from laboratory animals. Am J Trop Med Hyg.

[CR15] Edgar RC (2004). MUSCLE: multiple sequence alignment with high accuracy and high throughput. Nucleic Acids Res.

[CR16] Fedorko JM (1999). *Schistosoma japonicum* in the black rat, *Rattus rattus mindanensis*, from Leyte, Philippines in relation to *Oncomelania* snail colonies with reference to other endoparasites. Southeast Asian J Trop Med Public Health.

[CR17] Garba M, Dalecky A, Kadaoure I, Kane M, Hima K, Veran S, Gagare S, Gauthier P, Tatard C, Rossi JP, Dobigny G (2014). Spatial segregation between invasive and native commensal rodents in an urban environment: a case study in Niamey, Niger. PLoS One.

[CR18] Granjon L, Duplantier JM (2009) Les rongeurs de l’Afrique sahélo-soudanienne. Publications scientifiques du MNHN, Paris, 215p. (Faune et flore tropicales ; 43), IRD editions, Marseille, France

[CR19] Hanelt B, Mwangi N, Kinuthia JM, Maina GM, Agola LE, Mutuku MW, Steinauer ML, Agwanda BR, Kigo Mungai LBN, Loker ES, Mkoji GM (2010) Schistosomes of small mammals from the Lake Victoria Basin, Kenya: new species, familiar species, and implications for schistosomiasis control. Parasitology 137(7):1109–1118. 10.1017/S0031182010000041 10.1017/S0031182010000041 20380765

[CR20] Hima K, Houéménou G, Badou S, Garba M, Dossou HJ, Etougbétché J, Gauthier P, Artige E, Fossati-Gaschignard O, Gagaré S, Dobigny G, Dalecky A (2019). Native and invasive small mammals in urban habitats along the commercial axis connecting Benin and Niger, West Africa. Diversity.

[CR21] Houéménou G, Gauthier P, Etougbétché J, Badou S, Dossou HJ, Agossou D, Picardeau M, Dobigny G (2019). Pathogenic *Leptospira* in commensal small mammals from the extensively urbanized coastal Benin. Urban Sci.

[CR22] Kumar S, Stecher G, Tamura K (2016). MEGA7: Molecular evolutionary genetics analysis Version 7.0. Mol Biol Evol.

[CR23] Leulmi H, Socolovschi C, Laudisoit A, Houéménou G, Davoust B, Bitam I, Raoult D, Parola P (2014). Detection of *Rickettsia felis*, *Rickettsia typhi*, *Bartonella* species and *Yersinia pestis* in fleas (Siphonaptera) from Africa. PLoS Negl Trop Dis.

[CR24] Lockyer A, Olson P, Østergaard P, Rollinson D, Johnston D, Attwood S, Southgate V, Horak P, Snyder S, Le T (2003) The phylogeny of the Schistosomatidae based on three genes with emphasis on the interrelationships of *Schistosoma* Weinland, 1858. Parasitology 126:203–224 10.1017/s0031182002002792 12666879

[CR25] Martin-Alonso A, Houéménou G, Abreu-Yanes E, Valladares B, Feliu C, Foronda P (2016). *Bartonella* spp*.* in small mammals, Benin. Vector-Borne Zoonotic Dis.

[CR26] Moné H, Holtfreter MC, Allienne J-F, Mintsa-Nguema R, Ibikounlé M, Boissier J, Berry A, Mitta G, Richter J, Mouahid G (2015). Introgressive hybridizations of *Schistosoma haematobium* by *Schistosoma bovis* at the origin of the first case report of schistosomiasis in Corsica (France, Europe). Parasitol Res.

[CR27] Mouahid A, Théron A (1986). *Schistosoma bovis*: patterns of cercarial emergence from snails of the genera *Bulinus* and *Planorbarius*. Exp Parasitol.

[CR28] Mouahid A, Moné H, Arru E, Chassé JL, Théron A, Combes C (1987). Analyse comparative du rythme d’émission des cercaires de trois souches de *Schistosoma bovis*. Parassitologia.

[CR29] Mouahid A, Moné H, Chaïb A, Théron A (1991). Cercarial shedding patterns of *Schistosoma bovis* and *Schistosoma haematobium* from single and mixed infections of *Bulinus truncatus*. J Helminthol.

[CR30] Mouahid G, Idris MA, Verneau O, Théron A, Shaban MM, Moné H (2012). A new chronotype of *Schistosoma mansoni*: adaptive significance. Trop Med Int Hlth.

[CR31] Nei M, Kumar S (2000). Molecular evolution and phylogenetics.

[CR32] Pitchford RJ (1977). A check list of definitive hosts exhibiting evidence of the genus *Schistosoma* Weinland, 1858 acquired naturally in Africa and the Middle East. J Helminthol.

[CR33] Rollinson D, Southgate VR (1987) The genus *Schistosoma*: a taxonomic appraisal. *In* The biology of schistosomes. From genes to latrines. Rollinson D, Simpson AJG Eds, Academic Press, London pp. 446

[CR34] Savassi AES, Mouahid G, Lasica C, Mahaman SDK, Garcia A, Courtin D, Allienne JF, Ibikounlé M, Moné H (2020) Cattle as natural host for *Schistosoma haematobium* (Bilharz, 1852) Weinland, 1858 x *Schistosoma bovis* Sonsino, 1876 interactions, with new cercarial emergence and genetic patterns. Parasitol Res 119(7):2189–2205. 10.1007/s00436-020-06709-0 10.1007/s00436-020-06709-0 32468189

[CR35] Su J, Zhou F, Lu DB (2013). A circular analysis of chronobiology of *Schistosoma japonicum* cercarial emergence from hilly areas of Anhui, China. Exp Parasitol.

[CR36] Théron A (1989). Hybrids between *Schistosoma mansoni* and *S. rodhaini*: characterization by cercarial emergence rhythms. Parasitology.

[CR37] Théron A, Combes C (1983). Analyse génétique du rythme d’émergence des cercaires de *Schistosoma mansoni* par croisement de souches à pics d’émission précoces ou tardifs. C R Acad Sci Paris.

[CR38] WHO (2020) Schistosomiasis. Fact sheet (2 March 2020)

